# Cocci: Their Demonstration and Significance

**Published:** 1907-06-08

**Authors:** 


### Pathology in General Practice.

### COCCI: THEIR DEMONSTRATION AND SIGNIFICANCE.

(*Continued from page* 90.)

The streptococci occur in chains of varying length, and at one time were divided into different species, such as the *streptococcus longus*, the *streptococcus brevis*, the *streptococcus of erysipelas*, etc. It is now more usual to consider all as simply varieties of one group, as by growing them on different culture media one form may easily be changed into another, and at the same time may have its virulence increased or diminished. Streptococci are capable of producing many different lesions in the human subject, the general type being that of extending inflammations with or without the formation of pus. In the secretions of the mouth and in sputum, forms may often be demonstrated which do not seem to produce any lesions, but apparently under certain conditions, when any lowering of the vitality of the tissues takes place, they may take on a more important rôle and cause varying grades of inflammation of the throat and also other pathological conditions. In addition, they may be associated with peritonitis, puerperal septicæmia, septicæmia from post-mortem wounds and otherwise, cellulitis, suppuration in joints, meningitis, pleurisy, ulcerative endocarditis, lymphangitis, and they are also present as the causative agent in erysipelas. It is specially important to be able to diagnose them in sloughing conditions of the throat with the formation of false membranes, but in order to be certain that the case was not true diphtheria, cultures would have to be made as well as a mere direct examination of smears.

### Their Demonstration.

All that is required is to make smears from the pus or sero-purulent material, and stain these by any of the simple stains mentioned in the last article (April 13, 1907, page 30). Gram's stain may also be employed; they retain this, and very pretty specimens may be obtained by using any pink or red counter-stain to colour the tissues in order to show up the cocci. It is not necessary to go in detail into the question of the growth of these organisms on culture media; they grow with greater difficulty, have totally different appearances, and die out much more easily than the staphylococci.

### The Pneumococcus.

Closely allied to the streptococci is a coccus which has been variously known by the names of the pneumococcus, the diplococcus pneumoniæ, the diplococcus lanceolatus, and the streptococcus pneumoniæ. Best known by the name of Fraenkel's pneumococcus, this organism is of great importance, as it is the commonest cause of lobar or croupous pneumonia. Just as in the case of the streptococci, it is said to be often found in the nasal and mouth secretions of apparently healthy people, an increase of virulence with multiplication of numbers rapidly taking place together with an extension to the lungs when any circumstances arise to lower sufficiently the vitality of the individual. For example, an intoxicated man, unable to reach home, sleeps out on the damp ground; in many instances there results an attack of pneumonia. In addition to lobar pneumonia, the pneumococcus may occur either by itself or associated more commonly with other organisms in broncho-pneumonia and bronchitis. By direct extension from the lung, empyema, pericarditis, or endocarditis, may in turn appear, while purulent meningitis due to it is by no means uncommon, either as a primary condition or secondarily following definite pneumonia. Further, it has been found in purulent peritonitis, otitis media, liver abscesses, and in arthritis. Hence it will be seen that its distribution may be a wide one and its scope for doing serious damage very extensive. Morphologically the coccus can be fairly readily distinguished. It occurs either in pairs or in short chains, each individual having its free extremity pointed, its opposed surface flat, this giving it the appearance of a lancet, from whence its name lanceolatus is derived. In addition to this a very distinct capsule can generally be made out around them, as a rule showing up as an unstained areola or halo. To demonstrate them, make films in the ordinary way. Pus removed by an exploratory puncture from an empyema, for example, or sputum from a suspected case of pneumonia, may be stained with carbol-fuchsin for half a minute, then decolorised slightly by washing in hot or almost boiling water for a few seconds, dried, and mounted. This method shows up the capsules very well, especially in the organisms from the pure pus from the pleural cavity, while if the case is one of pneumonia, the prevailing germs present in the sputum will be diplococci, and in many the capsules will also be readily detected. If Gram's stain is also retained we may be tolerably certain that we are dealing with Fraenkel's pneumococcus. The pneumococcus grows with difficulty on culture media, special ones having to be adopted for its development, and it dies out very rapidly, much more quickly, for example, than its relation, the ordinary streptococcus.

### Micrococcus Tetragenus.

Another coccus that may be met with is what has been called the micrococcus tetragenus. It is another of the pus producers, and is common in the secretions from bronchiectatic cavities in the lungs, and also in gangrenous conditions of those organs. It sometimes forms ordinary abscesses, a small one under the finger nail, once examined, probably got from a post-mortem wound, showing a pure culture of this organism. It is easily recognised morphologically, as owing to the fact of its dividing in two planes at right angles to one another, it is generally seen in groups of four, or tetrads sometimes surrounded by a faintly stained capsule. Any of the ordinary simple staining methods may be employed to demonstrate it, and it also stains by Gram.

